# Two-year outcomes of fenestrated and branched stent grafts in patients ≤65 years: A bicenter retrospective study

**DOI:** 10.1016/j.jvscit.2026.102283

**Published:** 2026-04-29

**Authors:** Emilien Van Weydevelt, Ziyad Alrajhi, Cindy Vannier, Justine Mougin, Blandine Maurel, Adrien Kaladji

**Affiliations:** a CHU Rennes, Service de Chirurgie Vasculaire, Rennes, France; b CHU Nantes, Département de chirurgie cardiaque et vasculaire, Nantes, France; c INSERM UMR 1087/CNRS UMR 6291, L’Institut du Thorax, Université de Nantes, Nantes, France; d INSERM, U1099, Rennes, France; e Université de Rennes 1, Laboratoire de Traitement du Signal et de l’Image (LTSI), Rennes, France

**Keywords:** Thoracoabdominal aortic aneurysm, Complex abdominal aortic aneurysm, Endovascular aneurysm repair, Young patients, Treatment outcome, Fenestrated and branched endografts

## Abstract

**Objective:**

Fenestrated and branched endovascular aortic repair (F/BEVAR) is increasingly used to treat complex abdominal aortic aneurysms and thoracoabdominal aortic aneurysms (TAAAs) because of their minimally invasive nature compared with open repair. However, data on outcomes in young patients remain limited. The aim of this study was to report 24-month outcomes in patients ≤65 years of age who underwent F/BEVAR.

**Methods:**

This retrospective bicenter cohort study included consecutive patients treated for complex abdominal aortic aneurysm or TAAA with F/BEVAR between 2016 and 2023 at Nantes and Rennes University Hospitals. Preoperative, intraoperative, and postoperative data were prospectively collected. Data were compared between patients ≤65 years of age and those >65 years of age. Follow-up analyses were performed at 24 months.

**Results:**

Among the 254 patients included, 24.8% were aged ≤65 years (n = 63). In the preoperative assessment, patients ≤65 years more frequently had TAAA (44.4% vs 19.9%; *P* < .001), a history of aortic dissection (19.0% vs 4.2%; *P* < .001), and prior aortic surgery (43.0% vs 28.0%; *P* = .046). Primary technical success was similar between the groups (96.8% vs 94.2%; *P* = .632). Thirty-day all-cause mortality was 3.2% in patients ≤65 years, similar to that observed in patients >65 years (4.2%; *P* = .90). Rates of postoperative medical complications within 30 days were also comparable, including acute kidney injury (7.9% vs 8.4%) and paraplegia (1.6% vs 0%). Surgical complications were mainly access related (6.3% vs 4.7%), with no significant difference between groups (*P* = .57). At 2 years, rates of endoleaks, stent occlusion, and reintervention for any cause were similar between groups. However, endovascular reintervention for branch stent placement was more frequent in patients ≤65 years (30.2% vs 10.5%; *P* < .001). Twenty-four-month mortality was 13.0% (11.1% vs 13.6%; *P* = .620). A trend toward greater aneurysm sac regression was observed in younger patients (−10.1% vs −4.4%; *P* = .10).

**Conclusions:**

Overall, outcomes after F/BEVAR were similar in patients ≤65 years of age compared with older patients, despite more extensive aneurysmal disease at baseline. The need for close surveillance of target vessels in these patients is confirmed, consistent with the higher prevalence of underlying and progressive aortic dissection.

**Article Highlights**

• **Type of Research:** Retrospective bicenter observational cohort study
• **Key Findings:** In this bicenter cohort, patients ≤65 years undergoing fenestrated and branched endovascular aortic repair had similar 24-month clinical success, survival, and aneurysm sac evolution compared with older patients, despite more extensive baseline aortic disease, but experienced a higher rate of branch-related secondary reinterventions.
• **Take Home Message:** Fenestrated and branched endovascular repair provides safe and effective midterm outcomes in patients ≤65 years, comparable with those observed in older patients despite more complex aortic disease, at the expense of a greater need for branch-related secondary procedures. Strict long-term surveillance of target vessels is, therefore, mandatory.

Complex abdominal aortic aneurysms (CAAAs) and thoracoabdominal aortic aneurysms (TAAAs) represent a significant clinical challenge because of their progressive nature and rupture-related mortality. The risk of aneurysmal rupture increases with aneurysm size and is associated with a mortality rate of 80% to 90%.^1^

Historically managed with open surgery, these aneurysms are associated with substantial perioperative morbidity and mortality, particularly in elderly patients and those with significant comorbidities.^2^^,^^3^ Endovascular techniques have substantially modified treatment strategies over the past four decades. The introduction of fenestrated and branched endovascular aneurysm repair (F/BEVAR) has enabled the treatment of complex aneurysms while preserving visceral artery perfusion, offering a less invasive alternative to open repair.

Early and midterm outcomes have demonstrated high technical success rates and decreased perioperative mortality and morbidity with F/BEVAR compared with open surgery, especially in high-risk patients.^4^ As a result, complex endovascular repair has become a first-line option in specialized centers for patients deemed unfit for open surgery, provided anatomical feasibility.^5^

However, the survival benefit observed in elderly and fragile populations is often limited by nonaortic mortality. Concurrently, improvements in device technology and preoperative planning have expanded the indications for F/BEVAR to younger patients with low to intermediate surgical risk, raising questions about the long-term relevance of this strategy in such patients.^6^ In younger patients, the choice between open and endovascular repair remains debated: although F/BEVAR decreases perioperative morbidity and hospital stay, it appears to be associated with a higher rate of secondary reinterventions, particularly involving target vessels.^7^ Long-term durability data remain limited, because these techniques were initially developed for high-risk patients with limited life expectancy.

Moreover, younger patients frequently present with specific conditions such as diffuse aortic disease or a history of aortic surgery, which may adversely affect long-term endovascular outcomes and increase the risk of branch-related complications or secondary endoleaks, underscoring the need for prolonged surveillance.^6^^,^^8^

In this context, younger patients represent a relevant subgroup in whom durability and reintervention burden are critical considerations. Therefore, this study aimed to analyze the 24-month outcomes of F/BEVAR for CAAA and TAAA in patients aged ≤65 years and to compare them with those of older patients, to evaluate the safety, effectiveness, and implications for follow-up and reintervention strategies in this younger population.

## Methods

### Type of study and participating centers

This retrospective observational study was conducted at two university hospital centers (Nantes and Rennes) between January 2016 and November 2023. Both centers are primary care hospitals for aortic surgery, with recognized expertise in the management of complex aortic aneurysms, whether by open surgery or endovascular surgery using F/BEVAR.

### Study population

All consecutive patients who underwent FEVAR and/or BEVAR were included. Inclusion and exclusion criteria are detailed in Table I. The indications concerned complex aortic aneurysms (juxtarenal, pararenal, suprarenal, or thoracoabdominal), including chronic postdissection and degenerative aneurysms.

**Table I:** Inclusion and exclusion criteria

Inclusion criteria	Complex EVAR (fenestrated and/or branched endovascular aneurysm repair)
	Procedures performed between January 2016 and November 2023
Exclusion criteria	Age <18 years
	Open surgical repair only
	Hybrid procedures (open and endovascular)
	Repair of ruptured aortic aneurysms requiring emergency intervention

*EVAR*, Endovascular aortic repair.

### Group composition

Patients were divided into two distinct groups based on their age at the time of surgery. The study group included patients aged ≤65 years on the day of the procedure. The 65-year threshold, although arbitrary, was selected to distinguish populations with differing therapeutic priorities and durability concerns. Although the primary objective in older patients is to decrease perioperative morbidity and mortality, in younger patients the focus shifts toward long-term durability and the burden of secondary reinterventions. This approach is consistent with previous studies that have used age-based stratification to assess outcomes after complex EVAR, albeit with varying thresholds (eg, 70 years).^6^

### Organization of operating centers

In each of the two centers, procedures were performed under the supervision of a single lead surgeon who were experienced in this technique (B.M. in Nantes and A.K. in Rennes).

### Endovascular devices and surgical strategy

Preoperative planning was based on the analysis of a multiphase preoperative computed tomography angiography (CTA) with three-dimensional reconstruction using dedicated software: AW Server (GE Healthcare) in Nantes and Endosize (Therenva) in Rennes. This approach made it possible to define the dimensions of the stent graft, the position of the fenestrations and/or branches, and the implantation strategy. No artificial intelligence-based tools were used for aneurysm measurements or for the identification of collateral arteries. All measurements and anatomical assessments were performed manually using dedicated imaging software by experienced operators. The planning and design of custom-made stents were carried out according to a standardized process, validated by the lead surgeon. All custom-made devices were manufactured by Cook Medical.

The choice between a fenestrated and/or branched stent graft module was dictated mainly by the operator's preferences and the anatomy. Bridging stents were selected at the discretion of the operator among commercially available covered balloon-expandable or self-expanding devices, including Advanta V12 (Getinge), BeGraft (Bentley InnoMed), and Viabahn (W. L. Gore & Associates).

Almost all procedures were performed under general anesthesia (except in selected patients with severe respiratory impairment). A surgical or percutaneous vascular approach was used depending on the patient's anatomy. Systemic anticoagulation with unfractionated heparin was administered intraoperatively and then monitored during the procedure to maintain activated clotting time according to institutional protocol.

### Data collection

Data were prospectively collected in each center using local institutional databases dedicated to patients undergoing F/BEVAR. These databases were independently maintained in Nantes and Rennes and subsequently merged for the purpose of the present retrospective analysis. The data collected included demographic parameters (age, sex), associated risk factors and comorbidities, aneurysm anatomical characteristics, procedural data, intraoperative and postoperative outcomes, and events occurring during follow-up and until 24 months postoperatively. Missing data were not imputed.

### Postoperative follow-up

Postoperative follow-up was similar between the two centers, standardized, and in accordance with best practice recommendations (European Society for Vascular Surgery 2024^5^). An initial follow-up consultation was scheduled between 1 and 3 months postoperatively, combined with a CTA. Further follow-up was then organized 1 year postoperatively with a CTA, then annually in the absence of intercurrent events, supplemented by an annual imaging examination (CTA and/or Doppler ultrasound examination). The decision to use Doppler ultrasound monitoring was made on a case-by-case basis in selected patients (no obesity, previous CTA showing no complication) or in the presence of contraindications to CTA (severe chronic renal failure).

### End points and definitions

Outcomes were assessed at 30 days, 12 months, and 24 months after the index procedure. The primary end point was 2-year clinical success in patients aged ≤65 years, whereas early (30-day) and intermediate (12-month) outcomes were considered secondary analyses.

Clinical success was defined according to the Society for Vascular Surgery reporting standards for the repair of complex aortic aneurysms, as successful deployment and implantation of all device components, with continued aneurysm exclusion, absence of aneurysm-related death, absence of type I or III endoleak, no aneurysm rupture, no conversion to open repair, no device migration or component separation, and no need for secondary intervention during follow-up. Secondary end points included 30-day and 2-year all-cause mortality, freedom from stent graft-related secondary intervention, freedom from target vessel instability, freedom from type I or III endoleak, aneurysm sac behavior (stability, regression, or enlargement >10 mm), and the rate and severity of postoperative complications, graded according to the Clavien-Dindo classification (with major complications defined as grade III or higher).

Secondary interventions were defined as any unplanned endovascular or open procedure performed after the index fenestrated/branched repair to maintain or restore aneurysm exclusion, device integrity, or target vessel patency, including treatment of type I or III endoleak, device migration, component separation, aneurysm sac enlargement requiring reintervention, or target vessel stenosis or occlusion requiring angioplasty, relining, restenting, thrombectomy, or thrombolysis. Type II endoleaks were treated only in the presence of aneurysm sac enlargement of >10 mm, in accordance with current Society for Vascular Surgery/European Society for Vascular Surgery recommendations. Access-related complications requiring reintervention were excluded.

Postoperative complications were graded according to the Clavien-Dindo classification.^9^ Acute renal failure was defined according to the Kidney Disease: Improving Global Outcomes (KDIGO) criteria and was included as a postoperative complication.^10^ Target vessel instability was defined as the occurrence of target vessel occlusion, significant stenosis, or the need for a target vessel-related secondary reintervention during follow-up.

### Ethical and regulatory aspects

Written information on the use of data for research purposes was made available to patients on the University Hospital Center website. The study was analyzed by the Rennes University Hospital Center research and innovation department, which classified it as noninterventional research. All procedures and devices used are part of standard clinical practice. Consequently, and in accordance with Article L. 1121-1 of the Public Health Code (Jardé Law), the study is classified at level three (RIPH 3) and does not require the collection of informed consent from patients. This study was approved to the Institutional Review Board of Rennes University Hospital Center.

### Statistical analyses

Continuous variables are reported as mean ± standard deviation or median (interquartile range [IQR]), as appropriate. Comparisons between groups were performed using Pearson's χ^2^ test or Fisher's exact test, depending on the expected numbers. Comparisons are reported as odds ratios (OR) with their 95% confidence intervals (CI). The threshold for statistical significance was set at a *P* value of <.05.

Survival, reintervention, and target vessels instability analyses were performed using the Kaplan-Meier method with comparison of curves using the log-rank test. Kaplan-Meier estimates are presented with standard error of <10%. Multivariate analyses including logistic regression and Cox regression models were used to identify factors associated with early and late mortality. Although longer follow-up was available for a proportion of patients, the 24-month time point was selected to allow homogeneous comparison between groups and to limit attrition bias.

Given the sample size and the exploratory nature of the study, no propensity matching or risk adjustment between age groups was performed. All statistical analyses were performed using RStudio software (The R Foundation for Statistical Computing). No a priori sample size calculation was performed.

## Results

### Study population and follow-up

Among the 254 patients included, 63 (24.8%) were aged ≤65 years. The median follow-up duration was 60.9 months (IQR, 32.8-96.0 months), with no significant difference between patients ≤65 years and those >65 years (59.8 months [IQR, 36-91.2 months] vs 61 months [IQR, 32.6-96 months]; *P* = .835). Baseline demographic and aneurysm characteristics are reported in Table II. Procedural characteristics and target vessel management are shown in Tables III and IV.

**Table II:** Patient characteristics

Variable	Global	≤65 years old	>65 years old	*P* value^a^
Patients	254 (100)	63 (24.8)	191 (75.2)	
Age at the time of the procedure, years	71 (66-78)	61 (58-64)	74 (70-80)	<.001
Sex				
Male	190 (74.8)	49 (77.8)	141 (73.8)	.646
Female	64 (25.2)	14 (22.2)	50 (26.2)	
Surgical indication				
Chronic postaortic dissection aneurysm	15 (5.9)	6 (9.5)	9 (4.7)	.388
Degenerative aneurysm	239 (94.1)	57 (90.5)	182 (95.3)	
Type of aneurysm				
CAAAs	188 (74.0)	35 (55.6)	153 (80.1)	<.001
TAAAs	66 (26.0)	28 (44.4)	38 (19.9)	
Comorbidities				
BMI (kg/m^2^)	26.4 (24.0-30.0)	27.7 (24.95-30.7)	26.2 (24.0-29.9)	.202
Obesity (BMI ≥30)	68 (26.8)	20 (31.7)	48 (25.1)	.387
Hypertension	202 (79.5)	52 (82.5)	150 (78.5)	.615
Diabetes	46 (18.1)	15 (23.8)	31 (16.2)	.244
Dyslipidemia	168 (66.1)	39 (61.9)	129 (67.5)	.505
Smoking (current or former)	164 (64.6)	46 (73.0)	118 (61.8)	.143
Alcohol consumption	27 (10.6)	11 (17.5)	16 (8.4)	.073
Cardiac arrhythmia	44 (17.3)	8 (12.7)	36 (18.9)	.354
Ischemic heart disease	112 (44.1)	26 (41.3)	86 (45.0)	.708
Chronic renal failure (eGFR <60 mL/min/1.73 m^2^)	37 (14.6)	9 (14.3)	28 (14.7)	.900
Peripheral arterial disease	40 (15.7)	14 (22.2)	26 (13.6)	.153
History of aortic dissection	20 (7.9)	12 (19.0)	8 (4.2)	<.001
History of aortic surgery	81 (31.9)	27 (42.9)	54 (28.3)	.046
Previous treatments				
Curative anticoagulation	47 (18.5)	10 (15.9)	37 (19.4)	.665
Single antiplatelet therapy	149 (58.7)	37 (58.7)	112 (58.6)	.476
Dual antiplatelet therapy	10 (3.9)	4 (6.3)	6 (3.1)	
ASA class				
1-2	54 (21.3)	11 (17.5)	43 (22.5)	.529
3	175 (68.9)	44 (69.8)	131 (68.6)	
4-5	25 (9.8)	8 (12.7)	17 (8.9)	
RCRI score				
1	56 (22.0)	16 (25.4)	40 (20.9)	.857
≥2	198 (78.0)	47 (74.6)	151 (79.1)	

*ASA*, American Society of Anesthesiologists; *BMI*, body mass index; *CAAAs*, complex abdominal aortic aneurysms; *eGFR*, estimated glomerular filtration rate; *RCRI*, Revised Cardiac Risk Index; *TAAAs*, thoracoabdominal aortic aneurysms.

Values are reported as number (%) or median (interquartile range), as appropriate.

a Categorical variables were compared using Fisher's exact test when appropriate.

**Table III:** Procedural characteristics

Variable	Global	≤65 years old	>65 years old	*P* value^a^
Procedure status				
Planned	242 (95.3)	58 (92.1)	184 (96.3)	.297
Urgent	12 (4.7)	5 (7.9)	7 (3.7)	
Type of procedure				
FEVAR	233 (91.7)	57 (90.5)	176 (92.1)	.295
BEVAR	20 (7.9)	5 (7.9)	15 (7.9)	
F/BEVAR	1 (0.4)	1 (1.6)	0	
No. of fenestrations/branches				
1-2	12 (4.7)	2 (3.2)	10 (5.2)	.037
3-4	234 (92.1)	56 (88.9)	178 (93.2)	
≥5	8 (3.2)	5 (7.9)	3 (1.6)	
Access				
Percutaneous femoral	159 (62.6)	45 (71.4)	114 (59.7)	.085
Surgical femoral (any)	77 (30.3)	17 (27.0)	60 (31.4)	
Combined access (axillary and femoral)	18 (7.1)	1 (1.6)	17 (8.9)	

*BEVAR*, branched endovascular aortic repair; *F/BEVAR*, fenestrated and branched endovascular aortic repair; *FEVAR,* fenestrated endovascular aortic repair.

Values are reported as number (%).

a Categorical variables were compared using Fisher's exact test when appropriate.

**Table IV:** Target vessel management

Variable	Global	≤65 years old	>65 years old	*P* value^a^
Celiac trunk				
Occlusion	12 (4.7)	3 (4.8)	9 (4.7)	.990
Fenestration	219 (86.2)	54 (85.7)	165 (86.4)	
Branch	23 (9.1)	6 (9.5)	17 (8.9)	
Superior mesenteric artery				
Occlusion	2 (0.8)	1 (1.6)	1 (0.5)	.570
Fenestration	229 (90.2)	55 (87.3)	174 (91.1)	
Branch	23 (9.0)	7 (11.1)	16 (8.4)	
Left renal artery				
Occlusion	10 (3.9)	4 (6.3)	6 (3.1)	.243
Fenestration	231 (90.9)	54 (85.7)	177 (92.7)	
Branch	13 (5.1)	5 (7.9)	8 (4.2)	
Right renal artery				
Occlusion	14 (5.5)	2 (3.2)	12 (6.3)	.202
Fenestration	229 (90.2)	56 (88.9)	173 (90.6)	
Branch	11 (4.3)	5 (7.9)	6 (3.1)	
Accessory renal arteries (left and right)				
Absence	215 (84.6)	51 (80.9)	164 (85.9)	.043
Embolization	31 (12.2)	7 (11.1)	24 (12.5)	
Stenting	8 (3.2)	5 (8)	3 (1.6)	

Values are reported as number (%). Detailed target vessel management is provided in Supplementary Table I (online only).

a Categorical variables were compared using Fisher's exact test when appropriate.

#### Primary end point

At 24 months of follow-up, clinical success did not differ significantly between patients aged ≤65 years and those >65 years (88.9% vs 89.5%; *P* = .99). No significant differences were observed between the groups in the occurrence of events constituting clinical success, including all-cause mortality, secondary procedure-related reinterventions, target vessel events, type I or III endoleaks, aneurysmal rupture, open surgical conversion, stent migration, or endograft disconnection during follow-up.

### Secondary end points

#### All-cause mortality

**Fig 1:**
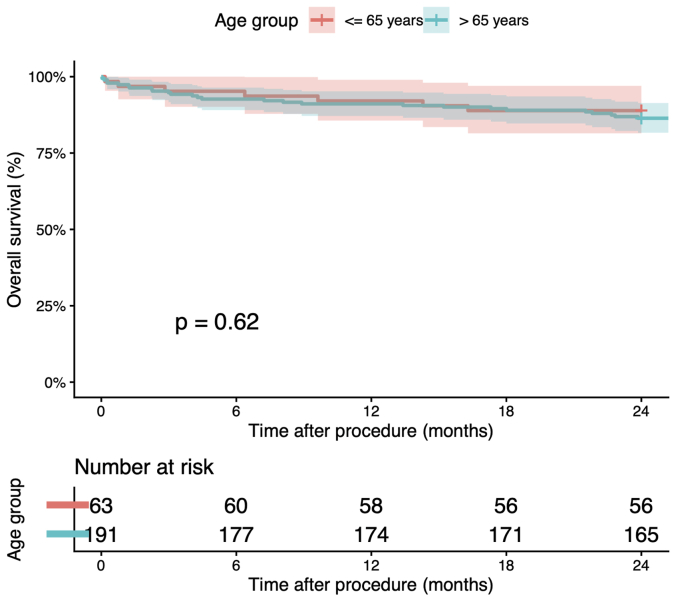
Overall survival at 24 months

All-cause mortality at 30 days was 3.9% (n = 10), with no significant difference between patients ≤65 years and those >65 years (3.2% vs 4.2%; *P* = .9). At 24 months, all-cause mortality reached 13% (33/254), with no significant difference according to age (11.1% ≤65 years vs 13.6% >65 years; *P* = .90). Overall survival are shown in Fig 1.

#### Technical success and early clinical success

Primary technical success was achieved in 94.9% of patients, with no significant difference between the age groups (*P* = .63). Primary clinical success at 30 days was 89.4% in the overall population, with comparable rates between the two groups (*P* = .99). Assisted clinical success reached 97.6% in the overall cohort, with no difference according to age (96.8% in patients ≤65 years vs 97.9% in patients >65 years; *P* = .99).

#### Reintervention

At 2 years, a total of 72 patients (28.5%) required at least one secondary unplanned reintervention, with similar rates among patients ≤65 years and those >65 years. Early reinterventions (between 30 and 90 days) occurred in 9.8% of patients, with no significant difference according to age (14.3 vs 8.4%; *P* = .262). Late reinterventions (>90 days) occurred in 15.3% of patients, also with no significant difference between groups (17.5% vs 14.7%; *P* = .739). Late results are shown in Table V.

**Table V:** Late results

Variable	Global	≤65 years old	>65 years old	*P* value^a^
Follow-up duration, months	60.9 (32.8-96)	59.8 (36-91.2)	61 (32.6-96)	.835
All-cause mortality at 24 months	33 (13)	7 (11.1)	26 (13.6)	.620
All-cause mortality at last follow-up	52 (20.5)	9 (14.3)	43 (22.5)	.221
Target vessels	60 (23.6)	18 (28.6)	42 (22)	.370
CAAA group	36 (14.2)	5 (7.9)	31 (16.2)	.485
TAAA group	24 (9.4)	13 (20.6)	11 (5.8)	.197
Complications				
Endoleaks	85 (33.5)	18 (28.6)	67 (35.1)	.426
Type IA	2 (0.8)	0	2 (1)	
Type IB	13 (5.1)	2 (3.2)	11 (5.8)	
Type IC	8 (3.1)	5 (7.9)	3 (1.6)	
Type II	48 (18.9)	11 (17.5)	37 (19.3)	
Type III	17 (6.7)	3 (4.8)	14 (7.3)	
Branch occlusion	26 (10.2)	8 (12.7)	18 (9.4)	.614
Occlusion of other vessels	3 (1.2)	2 (3.2)	1 (0.5)	
Reinterventions				
30-90 days	25 (9.8)	9 (14.3)	16 (8.4)	.262
>90 days	39 (15.3)	11 (17.5)	28 (14.7)	.739
Total				
1	48 (19)	12 (19)	36 (18.8)	.259
2	17 (6.7)	8 (12.7)	9 (4.7)	
3	6 (2.4)	1 (1.6)	5 (2.6)	
4	1 (0.4)	0	1 (0.5)	
Type of reinterventions				
Endovascular	39 (15.3)	19 (30.2)	20 (10.5)	<.001
Open	7 (2.8)	1 (1.6)	6 (3.1)	

*CAAA*, Complex abdominal aortic aneurysm; *TAAA,* thoracoabdominal aortic aneurysm.

Detailed branch occlusions, and reintervention procedures are provided in Supplementary Table III (online only).

Values are reported as number (%) or median (interquartile range), as appropriate.

a Categorical variables were compared using Fisher's exact test when appropriate.

**Fig 2:**
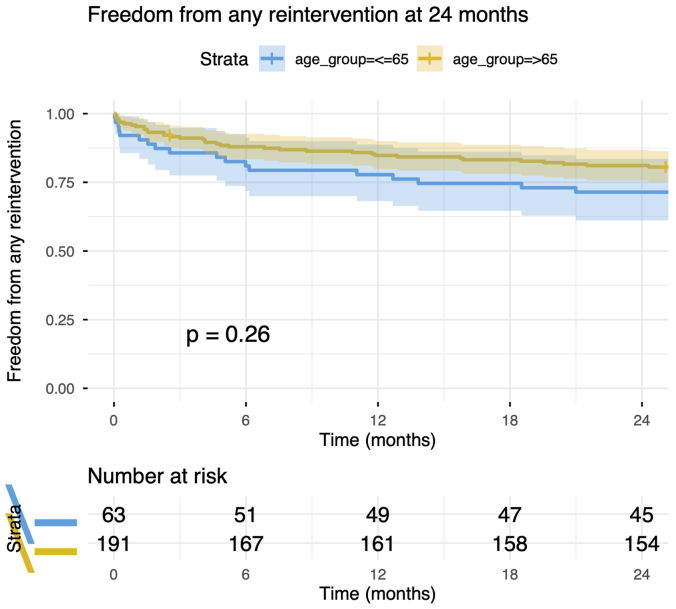
Freedom from any reintervention at 24 months.

The majority of reinterventions were performed via endovascular surgery (84.8%), with a significantly higher rate in patients ≤65 years of age, mainly for branch-related complications. Open surgical reinterventions remained rare (15.2% of reinterventions, or 2.8% of cases) and comparable between groups. See Supplementary Table I (online only) for detailed reintervention types. Freedom from any reintervention is shown in Fig 2.

### Target vessel events

Target vessel events, defined as occlusion, significant stenosis, or the need for a target-vessel-related secondary reintervention during follow-up, occurred in 23.6% of patients (28.6% vs 22%; *P* = .37). No statistically significant differences were observed according to aneurysm extent: in the TAAA group, 20.6% of patients ≤65 years vs 5.8% of patients >65 years (*P* = .197); and in the CAAA group, 7.9% of patients ≤65 years vs 16.2% of patients >65 years (*P* = .485). These subgroup comparisons should nevertheless be interpreted cautiously given the limited number of events. Detailed target vessel management is presented in Supplementary Table II (online only).

### Endoleaks

Endoleaks were observed in 33.5% of patients during follow-up, with no significant difference between age groups (28.6% vs 35.1%; *P* = .43). Endoleaks were predominantly type II (18.9%), followed by type III (6.7%), type IB (5.1%), IC (3.1%), and type IA (0.8%). At 2 years, 84.3% of patients had neither type I nor type III endoleaks, with no significant difference between the groups.

### Aneurysm sac evolution

At 2 years postoperatively, the mean aneurysm diameter continued to decrease (57.4 ± 16.5 mm), with numerically smaller diameters in patients aged ≤65 years (54.2 ± 14.2 mm) compared with those aged >65 years (58.4 ± 17.1 mm); however, this difference did not attain statistical significance (*P* = .11). Overall, no significant differences in aneurysm diameter evolution were observed between age groups throughout follow-up, suggesting comparable morphological remodeling after complex EVAR. AAA diameters are detailed in Table VI.

**Table VI:** Abdominal aortic aneurysm (*AAA*) diameter

AAA diameter, mm	Overall size	≤65 Years	>65 Years	*P* value
Preoperative	60.9 ± 9.3	60.3 ± 7.3	61.1 ± 9.9	.534
1 Month postoperative	60 ± 10.3	59.6 ± 7.9	60.2 ± 10.9	.666
1 Year postoperative	57.8 ± 13.3	56.9 ± 10.2	58.1 ± 14.2	.538
2 Years postoperative	57.4 ± 16.5	54.2 ± 14.2	58.4 ± 17.1	.109

Values are reported as mean ± standard deviation.

### Early postoperative complications (≤30 days)

Minor complications (Clavien-Dindo grades I-II), occurred in 20.5% of patients, with no significant difference according to age (19.0% vs 20.9%; *P* = .86). Postoperative acute renal failure was the most common complication. KDIGO 1 or 2 or KDIGO 3 without dialysis, classified as Clavien-Dindo grade II, affected 9% of patients, with no significant difference between the age groups (7.9% vs 8.4%). Among patients who developed acute postoperative renal failure, two (0.8%) required weekly dialysis sessions, and six patients (2.4%) showed partial recovery of renal function in the medium term. Complete recovery of renal function was observed in the other patients concerned (n = 15 [5.9%]). Early postoperative data are summarized in Table VII. Other minor complications are detailed in Supplementary Table III (online only).

**Table VII:** Early postoperative data

Variable	Global	≤65 Years	>65 Years	*P* value^a^
No. of days in intensive care	2.8 ± 5.7	3.16 ± 5.3	2.66 ± 5.83	.530
Length of hospital stay, days	8 ± 7.1	8.95 ± 7.94	7.69 ± 6.74	.263
30-Day all-cause mortality	10 (3.9)	2 (3.2)	8 (4.2)	.900
Primary technical success	241 (94.9)	61 (96.8)	180 (94.2)	.632
Primary clinical success	227 (89.4)	56 (88.9)	171 (89.5)	.990
Assisted primary clinical success	248 (97.6)	61 (96.8)	187 (97.9)	.990
Minor complications (Clavien-Dindo grade I-II)	52 (20.5)	12 (19)	40 (20.9)	.860
Major complications (Clavien-Dindo grade ≥ III)	9 (3.5)	2 (3.2)	7 (3.7)	.990
Complications requiring surgical treatment	25 (9.8)	8 (12.7)	17 (8.9)	.526

Values are reported as number (%) or mean ± standard deviation, as appropriate. Details of early complications are provided in Supplementary Table II (online only).

a Categorical variables were compared using Fisher's exact test when appropriate.

Major systemic complications (Clavien-Dindo ≥III) remained rare, occurring in 3.5% of patients, with no significant difference between age groups (3.2% vs 3.7%; *P* = .99). These included cardiac ischemic events (1.2%), stroke (0.4%), one case of paraplegia (0.4%), KDIGO 3 acute renal failure requiring dialysis (0.8%), disseminated intravascular coagulation (0.4%), and esophageal perforation (0.4%). Complications requiring surgical reintervention during the hospital stay affected 9.8% of patients, with no significant difference according to age (12.7% vs 8.9%; *P* = .53). These reinterventions were mainly related to conversion from percutaneous approach, acute lower limb ischemia, or early visceral complications.

## Discussion

In this retrospective, bicenter observational study, we analyzed the outcomes of CAAA and TAAA management by comparing two distinct groups using an age threshold of 65 years over a 24-month postoperative period. Our results did not differ between the two groups in terms of early and 24-month mortality, major complications, and changes in aneurysm diameter during follow-up.

In younger patients, a higher rate of endovascular reinterventions targeting branches reinforces the hypothesis of a different anatomopathological context in this age group (more TAAA, history of dissections, and aortic interventions). This study shows that endovascular repair using F/BEVAR in patients ≤65 years of age has early and midterm results comparable with those observed in an older population, despite initially more extensive and complete aortic disease.

The 30-day survival rate (96.8%) and 24-month survival rate (88.9%) in subjects ≤65 years were high. These results are comparable with those reported in the studies by Ferrer et al^6^ and Guéroult et al,^8^ despite a higher proportion of TAAAs, history of aortic dissection, and prior aortic surgery in the younger patient group. These data reinforce the hypothesis that chronological age, taken independently, is not a major predictor of postoperative mortality after complex endovascular repair.^11^ This finding confirms the importance of a comprehensive preoperative assessment that takes into account the patient's general condition, aortic anatomy, and comorbidities (American Society of Anesthesiologists, Revised Cardiac Risk Index score).^12^

The rate of early major complications was 3.5% (acute renal failure, cardiac complications, neurological complications, access complications), which were comparable between groups and consistent with recent studies.^6^ The absence of excess risk observed in the group that was ≤65 years supports the role of F/BEVAR as a valid treatment option, despite these patients being eligible for open surgery.^6^^,^^7^

The primary technical and clinical success rates observed in our study were high, comparable between age groups and with the literature (96.2% primary technical success in the study by Ferrer et al^6^ and 96% in the study by Feridooni et al^11^).

An analysis of morphological changes revealed a trend toward gradual reduction of the aneurysmal sac during follow-up, which appeared to be more pronounced in younger patients, but not significantly so. These data support effective and durable aneurysm exclusion in the midterm, including in younger patients with complex anatomy: there does not appear to be any early morphological failure in this subpopulation. Furthermore, the reduction in the aneurysmal sac in the medium term is suggested in some studies as being a major prognostic factor for long-term survival.^13^^,^^14^

One of the major findings of this study is the difference in the anatomical and pathophysiological profile of patients in the ≤65 years group. Indeed, this population had significantly more extensive TAAA-type aneurysmal pathologies, a history of chronic aortic dissection, and a history of aortic surgery. These factors may partly explain the greater frequency of target artery events observed in younger patients in our study, which is consistent with the existing literature on the subject.^15^, ^16^, ^17^

A significant difference was observed between the two groups with regard to endovascular reinterventions specific to target vessels, even though the overall reintervention rates at 24 months between the two groups were not significantly different. These were predominantly branch relining procedures performed for type I or III endoleaks, which were responsible for aneurysmal growth.

However, these results should not be interpreted as a failure of the F/BEVAR procedure in young patients, but rather as a reflection of more complex, progressive aortic disease associated with an increased cumulative risk of reintervention during follow-up.^18^^,^^19^ In addition, these young patients are often subject to closer monitoring of their condition due to this marked progressive profile, sometimes leading to earlier detection and therefore earlier endovascular correction.

These data are consistent with those reported in recent series focusing on young patients with multiple aneurysms, showing higher rates of reintervention related to target vessels in the midterm after F/BEVAR, but with no significant impact on overall survival. This finding reinforces the importance of informing young patients, during the preoperative consultation, of the increased risk of secondary reintervention and the importance of prolonged follow-up.^20^^,^^21^

Our study suggests that complex endovascular repair using F/BEVAR is a credible alternative to open surgery in young patients with CAAA or TAAA, provided that the anatomy is favorable and the procedure is performed in a high-volume, expert center.^6^^,^^7^^,^^22^ The immediate benefits in terms of preoperative morbidity, functional recovery, and length of hospital stay must be balanced against the increased risk of secondary reintervention related to target vessels.

Our study confirmed that, in young patients, the main issue does not appear to be the immediate safety of the procedure, but rather the durability of the repair and the organization of strict long-term follow-up.^5^^,^^23^ Frequent monitoring using CTA and Doppler ultrasound examination appears to be essential, especially in patients with dissecting pathology or a history of aortic surgery. However, the need for lifelong imaging surveillance must also be weighed carefully in younger patients, particularly with regard to cumulative radiation exposure and contrast administration.

Our study has several limitations. Its retrospective nature exposes it to selection bias. The 24-month follow-up period is short relative to the life expectancy of young patients, making it impossible to fully assess the long-term durability of F/BEVAR. This study should be considered an exploratory midterm analysis. Because younger patients undergoing FEVAR remain uncommon in routine practice and the number of observed events was limited, no robust multivariable regression analysis could be performed. In addition, the 24-month follow-up does not allow firm conclusions regarding long-term durability, and a 5-year update will be needed.

Finally, the lack of a direct comparison with young patients treated with open surgery does not allow us to conclude that one strategy is superior to the other in this population. Shared decision-making remains essential in this population. Prospective, multicenter studies with prolonged follow-up at 5 or 10 years are necessary to better characterize the durability of F/BEVAR procedures in young patients, particularly those with chronic dissection or hereditary aortic disease. In the future, assessing the long-term outcome of target vessels and continuously improving endoprosthetic devices, combined with optimized follow-up, could improve outcomes in this population.

## Conclusions

In conclusion, this study shows that complex endovascular repair using F/BEVAR in patients ≤65 years of age offers early and medium-term results comparable with those observed in older patients, despite initially more complex aortic disease. However, the higher frequency of target vessel reinterventions in younger patients highlights the need for individualized and prolonged monitoring. F/BEVAR appears to be a safe and effective therapeutic strategy in young patients, provided that rigorous selection and appropriate long-term follow-up are ensured.

## Author Contributions

Conception and design: EV, AZ, AK

Analysis and interpretation: EV, JM, BM, AK

Data collection: EV, AZ, CV

Writing the article: EV, AZ, CV, AK

Critical revision of the article: JM, BM, AK

Final approval of the article: EV, AZ, CV, JM, BM, AK

Statistical analysis: EV

Obtained funding: Not applicable

Overall responsibility: EV

## Funding

None.

## Disclosures

B.M. reports proctoring activities for W. L. Gore & Associates and Cook Medical and consulting activities for Getinge. A.K. reports proctoring fees for Cook Medical and proctoring and speaker fees for Medtronic and Biotronik.

## References

[1] Van Der Vliet J.A., Boll A.P. (1997). Abdominal aortic aneurysm. Lancet.

[2] LeMaire S.A., Price M.D., Green S.Y., Zarda S., Coselli J.S. (2012). Results of open thoracoabdominal aortic aneurysm repair. Ann Cardiothorac Surg.

[3] Wang S., Wang C., Gao Y., Tian Y., Liu J., Wang Y. (2024). Risk factors of 30-day and long-term mortality and outcomes in open repair of thoracoabdominal aortic aneurysm. J Cardiothorac Surg.

[4] Latz C.A., Boitano L., Schwartz S. (2021). Mortality is high following elective open repair of complex abdominal aortic aneurysms. Eur J Vasc Endovasc Surg.

[5] Wanhainen A., Van Herzeele I., Bastos Goncalves F., ESVS Guidelines Committee (2024). European Society for Vascular Surgery (ESVS) 2024 clinical practice guidelines on the management of abdominal aorto-iliac artery aneurysms. Eur J Vasc Endovasc Surg.

[6] Ferrer C., Gallitto E., Borghese O. (2024). Long-term results of fenestrated and branched endovascular aneurysm repair for complex abdominal and thoracoabdominal aortic aneurysms in young and fit patients. J Vasc Surg.

[7] Tinelli G., Sica S., Sobocinski J. (2024). Long-term propensity-matched comparison of fenestrated endovascular aneurysm repair and open surgical repair of complex abdominal aortic aneurysms. J Endovasc Ther.

[8] Guéroult A.M., Bashir A., Azhar B. (2024). Long-term outcomes and durability of fenestrated endovascular aneurysm repair: a meta-analysis of time-to-event data. Eur J Vasc Endovasc Surg.

[9] Dindo D., Demartines N., Clavien P.A. (2004). Classification of surgical complications: a new proposal with evaluation in a cohort of 6336 patients and results of a survey. Ann Surg.

[10] Khwaja A. (2012). KDIGO clinical practice guidelines for acute kidney injury. Nephron Clin Pract.

[11] Feridooni T., Gordon L., Mahmood D.N. (2024). Age is not a sole predictor of outcomes in octogenarians undergoing complex endovascular aortic repair. J Vasc Surg.

[12] Li J., Zou P., Zhou Y. (2025). Advanced age is significantly associated with poor outcomes of thoracic endovascular aortic repair: a systematic review and meta-analysis. BMC Surg.

[13] Mesnard T., Sulzer T.A.L., Kanamori L.R. (2024). Aneurysm sac shrinkage at 1 year after fenestrated-branched endovascular aortic repair of complex aortic aneurysms offers mid-term survival advantage. J Vasc Surg.

[14] Esposito D., Kaartama T., Bastianon M., SABER study group (2025). One-year stable sac predicts worse overall survival and midterm outcomes compared with sac regression after complex abdominal aortic aneurysm repair with fenestrated and branched endografts. Eur J Vasc Endovasc Surg.

[15] O’Donnell T.F.X., Patel P.B., Marcaccio C.L. (2023). Outcomes of complex endovascular treatment of post-dissection aneurysms. Eur J Vasc Endovasc Surg.

[16] Mylonas S.N., Aras T., Dorweiler B. (2024). A systematic review and an updated meta-analysis of fenestrated/branched endovascular aortic repair of chronic post-dissection thoracoabdominal aortic aneurysms. J Clin Med.

[17] Ribé L., Ruiter Kanamori L., Schmid B.P. (2025). Outcomes of fenestrated-branched endovascular aortic repair of thoracoabdominal aortic aneurysms in patients with heritable thoracic aortic diseases. JTCVS Tech.

[18] Fargion A.T., Esposito D., Speziali S., Pulli R., Gallitto E., IMF&B Study Group (2023). Fate of target visceral vessels in fenestrated and branched complex endovascular aortic repair. J Vasc Surg.

[19] Kärkkäinen J.M., Tenorio E.R., Jain A. (2020). Outcomes of target vessel endoleaks after fenestrated-branched endovascular aortic repair. J Vasc Surg.

[20] Tachida A., Stafforini N., Singh N., Starnes B., Zettervall S.L. (2023). Reinterventions after physician-modified endovascular grafts for treatment of juxtarenal aortic aneurysms are non-detrimental to long-term survival. J Vasc Surg.

[21] Gaston B.T., Eagleton M.J. (2024). A nonsystematic review of the early, mid-term, and long-term outcomes for fenestrated and branched endovascular repair of thoracoabdominal aneurysms. JVS-Vasc Insights.

[22] Raulli S.J., Gomes V.C., Parodi F.E. (2024). Five-year outcomes of fenestrated and branched endovascular repair of complex aortic aneurysms based on aneurysm extent. J Vasc Surg.

[23] Pineda D.M., Phillips Z.M., Calligaro K.D. (2017). The fate of endovascular aortic aneurysm repair after 5 years monitored with duplex ultrasound imaging. J Vasc Surg.

