# Supplementary material for: Two-year outcomes of fenestrated and branched stent grafts in patients ≤65 years: A bicenter retrospective study
Source: J Vasc Surg Cases Innov Tech. 2026 Apr 29;12(4):102283. doi: 10.1016/j.jvscit.2026.102283 (PMC13235450; doi:10.1016/j.jvscit.2026.102283)
Supplement: Supplementary Tables [file mmc1.docx]

| **Table SI. Detailed branch occlusions and reintervention procedures** | | | |  |
| --- | --- | --- | --- | --- |
| **Variable** | **Global** | **≤ 65 years old** | **> 65 years old** |  |
|  |  |  |  |  |
| **Complications** | | | |  |
| ***Endoleaks (see Table 5 for overall incidence)*** | 85 (33.5 %) | 18 (28.6 %) | 67 (35.1 %) |  |
| ***Branch occlusion*** | 26 (10.2 %) | 8 (12.7 %) | 18 (9.4 %) |  |
| Coeliac trunk | 3 (1.2 %) | 0 | 3 (1.6 %) |  |
| Superior mesenteric artery | 5 (2 %) | 2 (3.2 %) | 3 (1.6 %) |  |
| Right renal artery | 12 (4.7 %) | 4 (6.3 %) | 8 (4.9 %) |  |
| Left renal artery | 7 (2.6 %) | 2 (3.2 %) | 5 (2.6 %) |  |
| ***Occlusion of other vessels*** | 3 (1.2 %) | 2 (3.2 %) | 1 (0.5 %) |  |
| Bifurcated endoprosthesis module | 1 (0.4 %) | 1 (1.6 %) | 0 |  |
| Right common iliac artery | 1 (0.4 %) | 1 (1.6 %) | 0 |  |
| Left common iliac artery | 1 (0.4 %) | 0 | 1 (0.5 %) |  |
| **Reinterventions** | | | |  |
| **Endovascular** | 39 (15.3 %) | 19 (30.2 %) | 20 (10.5 %) |  |
| Branch stenting / endoleak | 16 (6.3 %) | 9 (14.3 %) | 7 (3.7 %) |  |
| Embolization of inferior mesenteric artery on type II endoleak | 6 (2.4 %) | 3 (4.8 %) | 3 (1.6 %) |  |
| Branch stenting / occlusion | 5 (2 %) | 2 (3.2 %) | 3 (1.6 %) |  |
| Extension by iliac branch endoprosthesis / type IB endoleak | 2 (0.8 %) | 1 (1.6 %) | 1 (0.5 %) |  |
| Proximal extension via TEVAR | 5 (2 %) | 3 (2.8 %) | 2 (1 %) |  |
| **Open** | 7 (2.8 %) | 1 (1.6 %) | 6 (3.1 %) |  |
| Partial explantation / device infection with replacement by open surgery | 2 (0.8 %) | 1 (1.6 %) | 1 (0.5 %) |  |
| Ligation of lumbar arteries (EFII) by open surgery | 1 (0.4 %) | 0 | 1 (0.5 %) |  |
| Fogarty catheter iliac desobliteration / Acute ischemia | 3 (1.2 %) | 0 | 3 (1.6 %) |  |
| Femorofemoral cross-over by pass / Acute ischemia | 1 (0.4 %) | 0 | 1 (0.5 %) |  |
| *Values are reported as n (%).* | | | |  |

| **Table SII. Detailed target vessel management** | | | | |  |
| --- | --- | --- | --- | --- | --- |
| **Variable** | **Global** | **≤ 65 years old** | **> 65 years old** | **p-value*** |  |
|  |  |  |  |  |  |
| Patients n (%) | 254 (100 %) | 63 (24.8 %) | 191 (75.2 %) |  |  |
| **Target vessel characteristics** | | | | |  |
| ***Celiac trunk*** | | | | |  |
| No stenting | 91 (35.8 %) | 17 (27.0 %) | 74 (38.7 %) | 0.415 |  |
| 1 covered stent | 145 (57.1 %) | 41 (65.1 %) | 104 (54.5 %) |  |  |
| ≥ 2 covered stents | 5 (2.0 %) | 1 (1.6 %) | 4 (2.1 %) |  |  |
| 1 bare stent and 1 covered stent | 13 (5.1 %) | 4 (6.3 %) | 9 (4.7 %) |  |  |
| ***Superior mesenteric artery*** | | | | |  |
| No stenting | 10 (3.9 %) | 2 (3.2 %) | 8 (4.2 %) | 0.182 |  |
| 1 covered stent | 213 (83.9 %) | 53 (84.1 %) | 160 (83.8 %) |  |  |
| ≥ 2 covered stents | 8 (3.1 %) | 4 (6.4 %) | 4 (2.1 %) |  |  |
| 1 bare stent and 1 covered stent | 23 (9.1 %) | 4 (6.3 %) | 19 (9.9 %) |  |  |
| ***Left renal artery*** | | | | |  |
| No stenting | 10 (4.0 %) | 5 (7.9 %) | 5 (2.6 %) | 0.327 |  |
| 1 covered stent | 216 (85 %) | 49 (77.8 %) | 167 (87.4 %) |  |  |
| ≥ 2 covered stents | 16 (6.3 %) | 4 (6.3 %) | 12 (6.3 %) |  |  |
| 1 bare stent and 1 covered stent | 12 (4.7 %) | 5 (7.9 %) | 7 (3.7 %) |  |  |
| ***Right renal artery*** | | | | |  |
| No stenting | 14 (5.5 %) | 2 (3.2 %) | 12 (6.3 %) | 0.677 |  |
| 1 covered stent | 212 (83.5 %) | 53 (84.2 %) | 159 (83.1 %) |  |  |
| ≥ 2 covered stents | 14 (5.5 %) | 4 (6.3 %) | 10 (5.3 %) |  |  |
| 1 bare stent and 1 covered stent | 14 (5.5 %) | 4 (6.3 %) | 10 (5.3 %) |  |  |
| *Values are reported as n (%).* | | | | |  |
| **Categorical variables were compared using Fisher’s exact test when appropriate.* | | | | |  |

| **Table SIII. Detailed early intraoperative and postoperative complications** | | | |  |
| --- | --- | --- | --- | --- |
| **Variable** | **Global** | **≤ 65 years old** | **> 65 years old** |  |
|  |  |  |  |  |
| **Minor (Clavien-Dindo grade I-II)** | 52 (20.5 %) | 12 (19 %) | 40 (20.9 %) |  |
| Cardiac (Arrhythmia) | 8 (3.1 %) | 3 (4.8 %) | 5 (2.6 %) |  |
| Acute pulmonary edema | 4 (1.6 %) | 0 | 4 (2.1 %) |  |
| Acute renal failure KDIGO 1-2 or 3 without dialysis | 21 (9 %) | 5 (7.9 %) | 16 (8.4 %) |  |
| Acute pneumopathy | 11 (4.3 %) | 3 (4.8 %) | 8 (4.2 %) |  |
| Deep vein thrombosis | 2 (0.8 %) | 1 (0.9 %) | 1 (0.5 %) |  |
| Urinary tract infection | 4 (1.6 %) | 0 | 4 (2.1 %) |  |
| Reflex ileus | 2 (0.8 %) | 0 | 2 (1 %) |  |
| **Major (Clavien-Dindo grade ≥ III)** | 9 (3.5 %) | 2 (3.2 %) | 7 (3.7 %) |  |
| Cardiac (Ischemia) | 3 (1.2 %) | 0 | 3 (1.6 %) |  |
| Stroke | 1 (0.4 %) | 0 | 1 (0.5 %) |  |
| Paraplegia | 1 (0.4 %) | 1 (1.6 %) | 0 |  |
| Acute renal failure KDIGO 3 with dialysis | 2 (0.8 %) | 1 (1.6 %) | 1 (0.5 %) |  |
| Disseminated Intravascular Coagulation (DIC) | 1 (0.4 %) | 0 | 1 (0.5 %) |  |
| Esophageal perforation | 1 (0.4 %) | 0 | 1 (0.5 %) |  |
| ***Complications requiring surgical treatment*** | 25 (9.8 %) | 8 (12.7 %) | 17 (8.9 %) |  |
| Intraoperative conversion from percutaneous approach / closure system failure | 13 (5.2 %) | 4 (6.3 %) | 9 (4.7 %) |  |
| Evacuation of hematoma | 3 (1.2 %) | 1 (1.6 %) | 2 (1 %) |  |
| Acute limb ischemia | 7 (2.8 %) | 1 (1.6 %) | 6 (3.1 %) |  |
| Hemorrhagic shock / Intraoperative iliofemoral rupture | 4 (1.6 %) | 1 (1.6 %) | 3 (1.6 %) |  |
| Hepatic and splenic ischemia / endoprosthetic coverage | 1 (0.4 %) | 1 (1.6 %) | 0 |  |
| Mesenteric ischemia | 3 (1.2 %) | 0 | 3 (1.6 %) |  |
| *Values are reported as n (%).* | | | |  |
